# Autonomic function effects on risk of postoperative complications in gastrointestinal tumors: a retrospective cohort study

**DOI:** 10.3389/fonc.2026.1877633

**Published:** 2026-10-30

**Authors:** Chen Yi, Haoyang Li, Haifeng Wang, Danli Shen, Huafeng Pan, Zhiwei Jiang, Gang Wang

**Affiliations:** 1 Affiliated Hospital of Nanjing University of Chinese Medicine, Nanjing, Jiangsu, China; 2 Nanjing University of Chinese Medicine, Nanjing, Jiangsu, China

**Keywords:** autonomic nervous system, heart rate variability, postoperative complications, radical surgery of gastrointestinal tumors, surgical stress

## Abstract

**Aim:**

To investigate the predictive value of dynamic heart rate variability (HRV) changes during the perioperative period for complications following radical surgery for gastrointestinal tumors.

**Methods:**

This retrospective cohort study included 231 patients who underwent elective radical surgery for gastrointestinal tumors at the Affiliated Hospital of Nanjing University of Chinese Medicine between May 2022 and September 2025. Based on the occurrence of complications during postoperative hospitalization, they were divided into a complication group (n = 41) and a non-complication group (n = 190). All patients wore a long-term wearable dynamic monitoring device recording dynamic heart rate variability indicators from 24 hours preoperatively to postoperative day 3. Concurrent changes in inflammatory markers were also recorded. Univariate and multivariable logistic regression analyses were used to construct prediction models, and receiver operating characteristic curves were plotted to evaluate their predictive performance.

**Results:**

The root mean square of successive differences on postoperative day 3 was significantly lower in the complication group than in the non-complication group, while the low-frequency/high-frequency ratio was higher and recovered slowly. Among the inflammatory markers, interleukin-6, white blood cell, and C-reactive protein levels were significantly higher in the complication group. The combined model incorporating heart rate variability trajectory parameters and serum markers demonstrated the highest predictive value (area under the curve [AUC] = 0.832, 95% CI: 0.762–0.902, P < 0.001).

**Conclusion:**

Dynamic perioperative HRV monitoring can effectively reflect the state of autonomic function under surgical stress. By combining HRV with inflammatory markers, we established the model to identify the association between HRV and the risk of short-term postoperative complications, thereby facilitating the early identification and intervention of high-risk patients.

## Highlights

- Dynamic perioperative HRV monitoring via intelligent patch technology captures the complete autonomic trajectory in gastrointestinal tumor patients.
- Postoperative complications correlate strongly with autonomic dysfunction, characterized by sustained vagal suppression and relative sympathetic dominance.
- Integrating HRV parameters with inflammatory biomarkers demonstrates a strong association with postoperative complications.
- HRV emerges as a novel non-invasive digital biomarker, enabling risk-stratified and individualized management of postoperative complications.

## Introduction

The global burden of gastrointestinal cancer is substantial. According to GLOBOCAN 2022 data, a total of 2.89 million new cases of gastrointestinal cancer were reported, accounting for 14.5% of global cancer incidences (1). Radical surgery has been a cornerstone in the treatment of gastrointestinal cancer; however, postoperative complication rates remain high at 10%–30% (2–4), severely worsening treatment efficacy and patient quality of life (5, 6). Traditional risk assessments primarily rely on static preoperative data, which lack sensitivity to dynamic physiological changes during the perioperative period, making early warning of postoperative complications difficult (7), resulting in an urgent need for early warning indicators that dynamically reflect physiological reserve. Heart rate variability (HRV), as a non-invasive biomarker (8), dynamically and sensitively reflects physiological reserve and regulatory capacity through analysis of minute fluctuations in consecutive heart beat intervals (9, 10) and is closely related to perioperative autonomic nervous system status and stress-induced inflammatory levels (11).

In the early postoperative period after gastrointestinal tumor surgery, stressors such as surgical trauma and pain can trigger sympathetic nervous system excitation and parasympathetic inhibition, leading to decreased HRV. If HRV remains persistently low, it indicates impaired autonomic nervous function and an increased risk of complications. The underlying mechanism involves autonomic dysfunction interfering with physiological processes including, but not limited to, immune regulation and gastrointestinal motility, weakening the body’s stress reserve, and increasing susceptibility to complications. In perioperative management, various interventions have been proven to optimize patient prognosis by improving HRV. For example, Enhanced Recovery After Surgery (ERAS) protocols, through multimodal analgesia combined with early mobilization, can effectively improve postoperative HRV indicators and reduce inflammation levels. Minimally invasive surgery (12), electroacupuncture (13), and transcutaneous electrical acupoint stimulation (14) can also promote recovery by modulating the autonomic nervous system and improving HRV. However, previous studies have mostly been limited to single-point prediction of specific complications, lacking analysis of entire dynamic trajectories and multi-indicator synergistic monitoring, thereby limiting the timeliness and precision of early warning. Therefore, this study innovatively constructed a prediction model combining continuous HRV monitoring throughout the perioperative period with inflammatory markers, aiming to improve the accuracy of postoperative complication risk prediction.

In light of this, this study selected patients undergoing radical surgery for gastrointestinal tumors. These patients often experience autonomic nervous system dysfunction due to damage to abdominal autonomic nerves, as well as systemic stress and inflammatory responses, all of which contribute to characteristic dynamic changes in HRV. Therefore, this study aimed to systematically characterize perioperative HRV trajectory changes and evaluate their association with postoperative complications by reviewing data from patients who had undergone standardized monitoring.

## Materials and methods

### Patients

This retrospective cohort study was conducted using data from a departmental clinical database maintained by our surgical team at the Affiliated Hospital of Nanjing University of Chinese Medicine. Between May 2022 and September 2025, 240 consecutive patients who underwent elective radical surgery for gastrointestinal tumors were identified from this database. After screening, 2 cases were excluded due to cardiac insufficiency, 2 cases due to incomplete HRV data, and 5 cases due to palliative surgery. Ultimately, 231 patients were included. Based on the occurrence of complications during postoperative hospitalization (Clavien–Dindo grade ≥I), they were divided into a complication group (n = 41) and a non-complication group (n = 190). All patients wore a long-term wearable dynamic ECG monitor, recording dynamic HRV indicators. Baseline data were systematically collected for both groups, including gender, age, body mass index (BMI), history of underlying diseases (diabetes, hypertension, cerebral infarction), tumor pathological stage, operation duration, surgical site, etc.

### Inclusion and exclusion criteria

#### Inclusion criteria

(1) Preoperative examination confirmed a gastrointestinal tumor and the patient underwent elective radical gastrectomy or colectomy; (2) Age ≥ 18 years, regardless of sex; (3) Conforming to American Society of Anesthesiologists (ASA) classification I–III; (4) The patient or their authorized trustee provided informed consent and signed the informed consent form.

#### Exclusion criteria

(1) Presence of significant arrhythmia or non-sinus baseline rhythm; (2) Patients with implanted cardiac pacemakers or cardioverter-defibrillators; (3) Complicated by severe liver or kidney failure; (4) Presence of cognitive impairment or mental illness; (5) Allergy to materials related to the wearable device; (6) Emergency or palliative surgery; (7) HRV monitoring data completeness less than 80%.

### Outcomes

#### Heart rate variability monitoring indicators

A patch-type dynamic ECG recorder (CH-E31, Zhejiang Zhirou Technology Co., Ltd., Medical Device Registration Certificate No. 20212070574) was used to collect dynamic ECG data. This device is a Class II medical device approved by the National Medical Products Administration (NMPA), and its clinical sensitivity and validity have been established in previous studies (15). The patch was positioned on the left chest approximately one finger-breadth below the clavicle (second intercostal space), with a placement angle of 45° in male patients and 60° in female patients. Recording commenced 24 hours before surgery and continued for 72 hours postoperatively. Raw electrocardiographic signals were uploaded to a cloud-based platform, where motion-induced artifacts were automatically filtered by proprietary algorithms (**Figures 1**, **2**). Ectopic beats were automatically detected and tagged by the device’s built-in QRS recognition algorithm. Only recordings with ≥80% valid R–R intervals within any 24-hour period were retained, and data from subjects with more than one consecutive missing recording day were excluded from HRV analysis. To minimize confounding by circadian rhythms and physical activity, all HRV parameters were extracted during the daytime window of 08:00 to 22:00. Time-domain indices included the standard deviation of all normal-to-normal intervals (SDNN), the standard deviation of the average NN intervals over short-term segments (SDANN), the root mean square of successive differences between adjacent NN intervals (RMSSD), and the percentage of successive NN intervals differing by more than 50 ms (pNN50). Patients receiving beta-blockers, anticholinergic agents, or epidural anesthesia, and those requiring continuous perioperative opioid infusion were excluded from the final analysis to minimize the potential confounding effects of these medications on autonomic nervous system activity.

**Figure 1:**
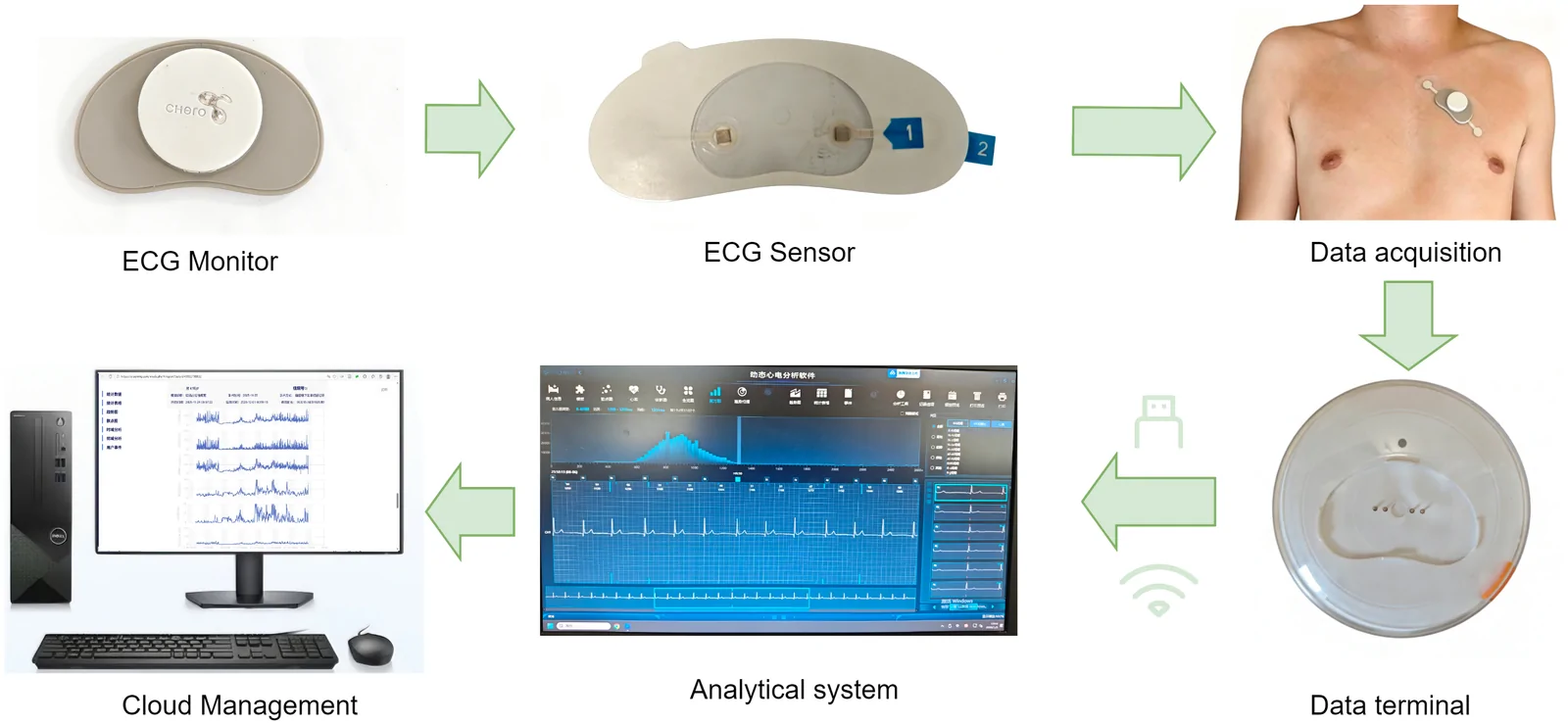
Patch-type dynamic HRV recorder.

**Figure 2:**
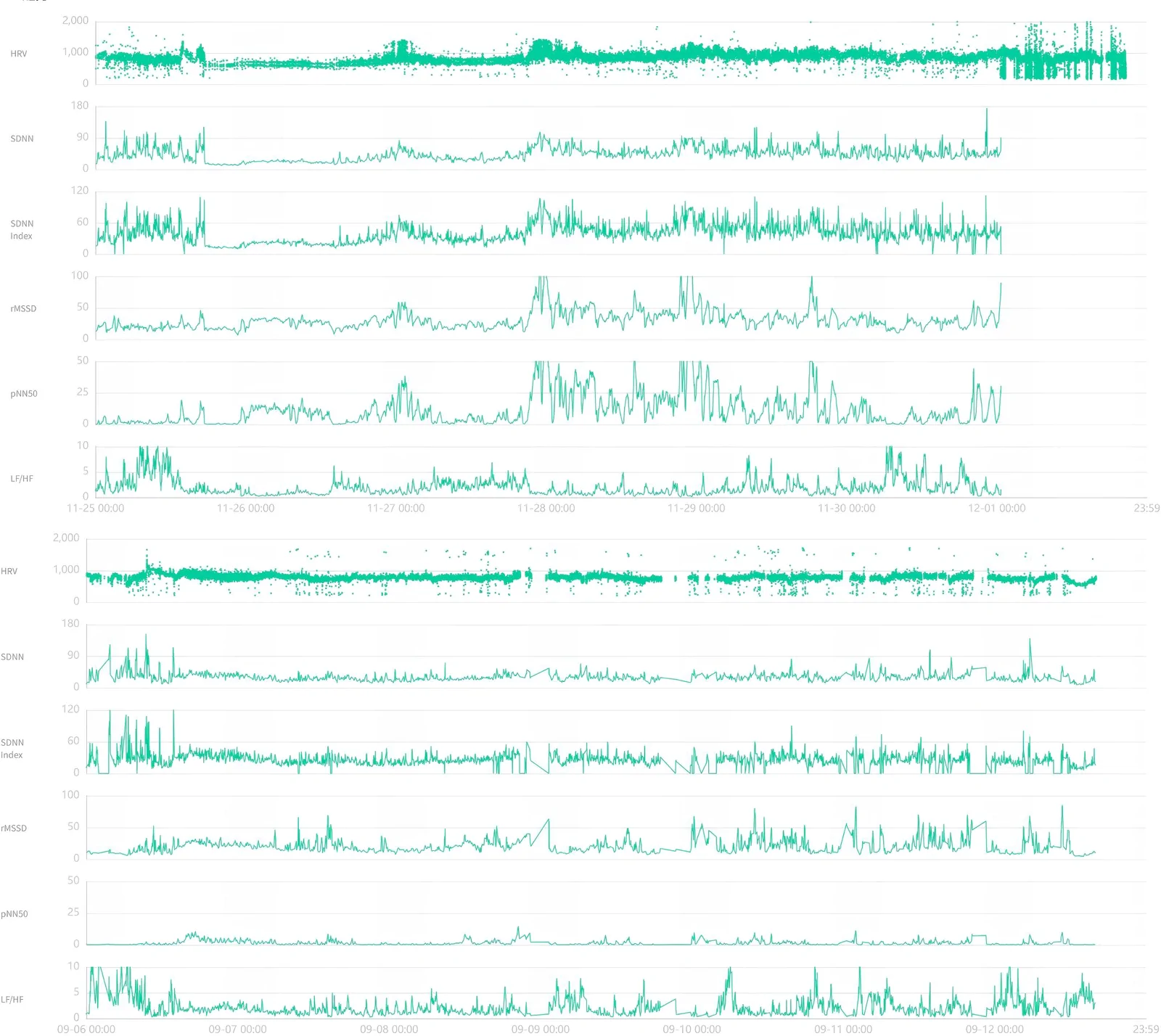
Trend charts of HRV during the perioperative period for patients in the non-complication group **(A)** and the complication group **(B)**.

#### Inflammatory indicators

Preoperative inflammatory biomarkers and those measured on postoperative days 1 and 3 were collected for both groups, including C-reactive protein (CRP), white blood cell count (WBC), interleukin-6 (IL-6), interleukin-2 (IL-2), and neutrophil percentage (NEUT%).

#### Postoperative complications

Postoperative complications for both groups were recorded according to the Clavien–Dindo classification (16). Grade I: requiring basic management or no specific intervention; Grade II: requiring pharmacological intervention but no invasive procedures; Grade III: requiring surgical, endoscopic, or radiological invasive intervention under local (IIIa) or general (IIIb) anesthesia; Grade IV: life-threatening organ failure requiring care in an intensive care unit (ICU), with single-organ failure as IVa and multi-organ failure as IVb; Grade V: death. Grades III, IV, and V were considered severe complications (17). Furthermore, diagnostic criteria for common postoperative complications of gastrointestinal tumors were defined: infectious complications such as pneumonia, urinary tract infection, wound infection, and abdominal infection were diagnosed according to the Hospital Infection Diagnostic Criteria (Trial) issued by the Ministry of Health of the People’s Republic of China. Pneumonia required abnormal imaging or positive bacterial culture; sepsis diagnosis followed the Sepsis-3 Consensus (18) (requiring a Sequential Organ Failure Assessment (SOFA) score ≥2). Circulatory complications like postoperative hypotension required the use of vasoactive drugs; respiratory complications like hypoxemia and acute respiratory failure required mechanical ventilation; surgery-specific complications such as anastomotic leak and chyle leak required imaging identification of the leak or clinical confirmation via puncture/drainage fluid, while others like intestinal obstruction and perforation required imaging confirmation; postoperative pain was assessed using the numeric rating scale (NRS). All complications were determined by two independent clinicians, with disagreements reviewed by a senior attending physician.

#### Statistical methods

Statistical analysis was performed using SPSS 27.0 and R software (version 4.5.2). Measurement data conforming to a normal distribution are expressed as mean ± standard deviation ( $\bar{x}$ ± s), and comparisons between groups were made using independent samples t-tests. Skewed distribution data are expressed as median (25th, 75th percentiles), and comparisons between groups were made using the Mann–Whitney U test. Count data are expressed as number (percentage), and comparisons between groups were made using the chi-square test. Univariate regression analysis was used to screen potential factors influencing postoperative complications. Multivariable logistic regression analysis was employed to identify independent risk factors. Internal validation was performed using bootstrap resampling (1,000 replicates) to calculate the optimism-corrected area under the curve (AUC) and calibration slope. The discriminative ability of the identified factors was evaluated using receiver operating characteristic (ROC) curves and the area under the curve. P < 0.05 was considered statistically significant.

This study is reported in accordance with the Strengthening the Reporting of Observational Studies in Epidemiology (STROBE) statement for cohort studies. In addition, the development and validation of the prediction model followed the Transparent Reporting of a Multivariable Prediction Model for Individual Prognosis or Diagnosis (TRIPOD) checklist. A completed STROBE checklist and TRIPOD guideline are provided as **Supplementary Tables**, respectively.

## Results

### Baseline

A total of 231 patients were included in the study: 190 in the non-complication group (110 male patients, 80 female patients; mean age 63.99 years; mean BMI 24.09 kg/m²) and 41 in the complication group (29 male patients, 12 female patients; mean age 65.95 years; mean BMI 24.03 kg/m²). Comparison of baseline clinical characteristics between the two groups showed no statistically significant differences (P > 0.05), indicating comparability (**Table 1**).

**Table 1:** The baseline features.

Variable	Event group (n = 41)	Normal group (n = 190)	t/χ^2^/Z value	P value
Age (years)	65.00 (58.00~73.50)	64.50 (58.00~72.00)	-0.821	0.410
Gender				
Male	29 (70.7%)	110 (57.9%)	2.319	0.128
Female	12 (29.3%)	80 (42.1%)		
BMI (kg/m²)	24.20 ± 3.24	24.09 ± 3.26	-0.212	0.832
History of abdominal surgery				
Yes	26 (63.4%)	138 (72.6%)	1.391	0.238
No	15 (36.6%)	52 (27.4%)		
History of underlying medical conditions				
Yes	18 (43.9%)	103 (54.2%)	1.437	0.231
No	23 (56.1%)	87 (45.8%)		
History of neoadjuvant chemotherapy				
Yes	7	35	0.041	0.839
No	34	155		
Surgery selection				
Robot	6 (14.6%)	17 (9.9%)	1.216	0.270
Laparoscopy	35 (85.4%)	173 (91.1%)		
Primary procedure				
Distal stomach	16 (39%)	57 (30.0%)	5.446	0.488
Proximal stomach	1 (2.4%)	14 (7.4%)		
Whole stomach	9 (22.0%)	52 (27.4%)		
Right hemi colon	3 (7.3%)	15 (7.9%)		
Left hemi colon	0 (0%)	10 (5.3%)		
Rectum	8 (19.5%)	27 (14.2%)		
Sigmoid colon	4 (9.8%)	15 (7.9%)		
Surgical time (min)	280.00 (235.00~360.00)	270 (230~315)	-1.237	0.216
Bleeding volume (mL)	50.00 (25.00~100.00)	50.00 (27.50~100.00)	-1.397	0.180
Preoperative levels of inflammatory factors				
CRP/ (mg/L)	1.78 (0.60~3.17)	1.37 (0.49~3.46)	-0.498	0.618
WBC/ (10^9^/L)	5.45 (4.21~7.43)	5.18 (4.36~6.59)	-0.399	0.690
NEUT%	56.60 (51.60~65.85)	61.55 (52.65~68.43)	-1.655	0.099
IL-2/pg/mL	2.18 (1.65~3.02)	2.08 (1.65~2.87)	-0.527	0.598
IL-6/pg/mL	3.32 (2.59~6.74)	3.78 (2.23~6.73)	-0.810	0.418
Preoperative HRV indicators				
SDNN/ms	107.60 (88.23~126.41)	102.74 (84.84~123.80)	-0.258	0.797
SDANN/ms	86.11 (60.22~106.34)	80.07 (61.33~99.68)	-0.858	0.391
RMSSD/ms	26.00 (19.61~33.33)	25.87 (17.16~38.49)	-0.853	0.394
pNN50/%	12.44 (9.23~19.49)	12.70 (2.65~17.69)	-1.543	0.123
LF/ms	676.24 (400.61~1198.79)	776.88 (339.31~1062.31)	-0.415	0.678
HF/ms^2^	663.43 (285.37~1070.22)	670.74 (185.25~990.73)	-0.928	0.354
LF/HF	1.22 (1.01~1.51)	1.33 (1.05~1.99)	-1.654	0.098

Data are presented in the form of mean ± standard deviation ( $\bar{X}$ ± s), median and interquartile range [M (IQR)], or percentage (%).

### Comparison of intestinal function recovery indicators

The time to first flatus, removal time of the drainage tube, and length of postoperative hospitalization were significantly shorter in the non-complication group compared to the complication group, with statistically significant differences (P < 0.05) (**Table 2**).

**Table 2:** Comparison of indicators for intestinal function recovery.

Indicators	Event group	Normal group	Z value	P value
Time to first flatus (h)	52.00 (37.69~70.00)	43.00 (30.00~54.56)	-2.761	0.006*
First liquid feeding time (h)	83.50 (44.45~137.24)	70.06 (46.41~95.13)	-1.349	0.177
First semi-liquid feeding time (h)	154.48 (121.42~235.30)	147.83 (123.43~192.22)	-1.344	0.179
Length of postoperative hospitalization (days)	12.46 (8.50~17.37)	7.00 (6.00∼9.00)	-5.895	<0.001**
Removal time of catheter (h)	19.00 (16.38~42.75)	18.50 (16.06~19.50)	-1.762	0.078
Removal time of drainage tube (days)	9.00 (5.93~16.00)	6.00 (4.00~9.00)	-2.482	0.013*

Compared with the Normal group, *P < 0.05. **P < 0.001.

Data are presented in the form of median and interquartile range [M (IQR)].

### Comparison of inflammatory indicators

On postoperative day 3, IL-6, WBC, and CRP levels were significantly higher in the complication group than in the non-complication group, with statistically significant differences (P < 0.05) (**Table 3**).

**Table 3:** Comparison of postoperative inflammatory markers.

Inflammatory markers	Event group	Normal group	Z value	P value
The first day after surgery				
CRP/ (mg/L)	40.93 (31.67~55.44)	33.31 (21.38~61.85)	-1.663	0.096
WBC/ (10^9^/L)	11.56 (9.51~15.40)	11.95 (9.99~14.36)	-0.240	0.811
NEUT%	87.20 (82.15~90.35)	88.40 (83.35~90.30)	-0.804	0.421
IL-2/pg/mL	2.42 (1.78~3.37)	2.31 (1.72~3.23)	-0.781	0.435
IL-6/pg/mL	26.47 (9.89~80.86)	15.55 (7.38~56.12)	-1.123	0.261
The third day after surgery				
CRP/ (mg/L)	73.01 (17.40~113.40)	30.48 (15.28~63.27)	-3.021	0.003*
WBC/ (10^9^/L)	8.17 (6.79~11.23)	7.45 (6.02~9.29)	-2.058	0.040*
NEUT%	77.20 (67.00~85.55)	73.80 (69.58~79.70)	-1.931	0.053
IL-2/pg/mL	2.12 (1.81~11.42)	2.35 (1.73~3.57)	-0.153	0.878
IL-6/pg/mL	5.45 (3.44~14.84)	4.68 (2.14~11.04)	-1.998	0.046*

Compared with the Normal group, *P < 0.05.

Data are presented in the form of median and interquartile range [M (IQR)].

### Comparison of HRV parameters

HRV parameters decreased significantly in both groups after surgery. On postoperative day 1, SDNN, RMSSD, pNN50, low-frequency (LF), and high-frequency (HF) levels in both groups were lower than preoperative levels, but inter-group differences were not statistically significant (P > 0.05). On postoperative day 3, the non-complication group had significantly higher RMSSD levels than the complication group (P < 0.001) and a significantly lower LF/HF ratio (P = 0.034) (**Table 4**).

**Table 4:** Comparison of HRV indicators.

HRV indicators	Event group	Normal group	Z value	P value
The first day after surgery				
SDNN/ms	66.39 (50.96~77.73)	58.98 (46.76~77.24)	-1.626	0.104
SDANN/ms	45.18 (35.42~65.90)	45.21 (31.43~60.96)	-0.706	0.480
RMSSD/ms	27.72 (18.65~39.70)	25.15 (19.24~33.27)	-0.812	0.417
pNN50/%	8.99 (3.60~10.12)	7.58 (2.75~11.73)	-0.191	0.849
LF/ms^2^	643.65 (400.87~838.57)	534.57 (297.29~871.08)	-1.013	0.311
HF/ms^2^	492.51 (206.38~790.08)	517.28 (237.50~803.06)	-0.113	0.910
LF/HF	1.30 (0.96~1.86)	1.13 (0.81~1.51)	-1.469	0.142
The third day after surgery				
SDNN/ms	71.57 (55.00~90.88)	71.32 (55.83~94.27)	-0.191	0.849
SDANN/ms	54.91 (36.94~76.31)	57.64 (44.91~83.75)	-1.245	0.213
RMSSD/ms	26.36 (15.12~31.51)	34.95 (28.38~46.30)	-5.339	<0.001**
pNN50/%	11.48 (3.74~14.97)	12.15 (5.02~16.07)	-0.917	0.359
LF/ms^2^	805.07 (287.63~1248.02)	808.62 (378.94~1091.26)	-0.374	0.709
HF/ms^2^	691.51 (225.34~935.55)	779.58 (319.13~1095.78)	-0.211	0.833
LF/HF	1.27 (0.94~1.40)	1.06 (0.81~1.29)	-2.116	0.034*

Compared with the Normal group, *P < 0.05. **P < 0.001.

Data are presented in the form of median and interquartile range (M (IQR)).

### Comparison of postoperative complications

Among the 231 patients undergoing gastrointestinal tumor surgery, 41 (17.7%) experienced short-term postoperative complications. Among the 41 patients with complications, the distribution by Clavien–Dindo grade was as follows: Grade ≤ II (n = 33, 80.5%), Grade III (n = 5, 12.2%), Grade IV (n = 3, 7.3%), and Grade V (n = 0). Severe complications (≥ III) accounted for 8 cases (19.5%), including 2 cases of anastomotic leak complicated by septic shock, 1 case of pulmonary embolism complicated by small bowel obstruction, 1 case of paralytic ileus with wound infection, 2 cases of duodenal stump leak, and 2 cases of anastomotic leak.

### Prediction model analysis

#### Model discrimination validation

Given the relatively low incidence of postoperative complications, a univariate screening threshold of P < 0.1 was adopted. Eight variables reached this threshold: CRP on postoperative day (POD) 1 and POD 3, WBC, neutrophil percentage, IL-2 on POD 3, preoperative pNN50, preoperative LF/HF, and POD 3 RMSSD (**Table 5**). Before entering these variables into the multivariable logistic regression model (**Table 6**), collinearity was assessed. The variance inflation factors (VIFs) were 1.323 for CRP on POD 1 and POD 3, and 1.173 for WBC and neutrophil percentage, both below the threshold of 5, indicating no severe multicollinearity. POD 3 RMSSD emerged as the strongest independent protective factor (odds ratio [OR] = 0.923, 95% CI: 0.888–0.960, P < 0.001), whereas POD 3 CRP was identified as an independent risk factor (OR = 1.012, 95% CI: 1.003–1.021, P = 0.008).

**Table 5:** Univariate analysis of risk factors.

Indicators	B	SE	Wald	Sig.	OR	95% confidence interval	
CRP on postoperative day 1	0.008	0.005	3.402	0.065	1.008	0.999	1.018
CRP on postoperative day 3	0.01	0.003	10.614	<0.001**	1.01	1.004	1.016
WBC on postoperative day 3	0.133	0.057	5.458	0.019*	1.143	1.022	1.278
NEUT% on postoperative day 3	0.034	0.019	3.118	0.077	1.035	0.996	1.075
IL-2 on postoperative day 3	0.039	0.017	5.1	0.024*	1.04	0.964	1.005
Preoperative pNN50	0.03	0.016	3.684	0.055	1.031	0.999	1.063
Preoperative LF/HF	-0.509	0.243	4.386	0.036*	0.601	0.374	0.968
RMSSD on postoperative day 3	-0.077	0.018	17.473	<0.001**	0.926	0.893	0.960

SE, Standard error; sig., level of significance; OR, odds ratio.

*P < 0.05. **P < 0.001.

**Table 6:** Binary multivariable logistic regression analysis of risk factors.

Indicators	B	SE	Wald	Sig.	OR	95% confidence interval	
CRP on postoperative day 1	-0.001	0.006	0.028	0.868	0.999	0.986	1.012
CRP on postoperative day 3	0.012	0.004	7.037	0.008*	1.012	1.003	1.021
WBC on postoperative day 3	0.081	0.075	1.173	0.279	1.084	0.937	1.255
NEUT% on postoperative day 3	0.004	0.021	0.033	0.856	1.004	0.964	1.045
IL-2 on postoperative day 3	0.021	0.018	1.263	0.261	1.021	0.985	1.058
Preoperative pNN50	0.035	0.023	2.241	0.134	1.035	0.989	1.084
Preoperative LF/HF	-0.530	0.290	3.325	0.068	0.589	0.333	1.040
RMSSD on postoperative day 3	-0.080	0.020	16.167	<0.001**	0.923	0.888	0.960
Constant	-0.409	1.640	0.062	0.062	0.664		

SE, Standard error; sig., level of significance; OR, odds ratio.

*P < 0.05. **P < 0.001.

ROC curve analysis (**Figure 3**) demonstrated that the combined model, based on the predicted probability from the multivariable regression, showed excellent discriminative ability (AUC = 0.832, 95% CI: 0.762–0.902, P < 0.001), outperforming POD 3 RMSSD alone (AUC = 0.766, 95% CI: 0.678–0.854, P < 0.001) and POD 3 CRP alone (AUC = 0.651, 95% CI: 0.546–0.755, P = 0.003). Internal validation using bootstrap resampling (1,000 replicates) yielded an optimism-corrected AUC of 0.792 (95% CI: 0.729–0.895). The complete regression formula of the combined prediction model was presented below: Logit(P)=−0.409−0.001×POD1 CRP + 0.012×POD3 CRP + 0.081×POD3 WBC + 0.004×POD3 NEUT%+0.021×POD3 IL2 + 0.035×Preoperative PNN50−0.530×Preoperative LFHF−0.080×POD3 RMSSD, where P indicated the risk probability of postoperative complications. The model exhibited good calibration (Hosmer–Lemeshow, P = 0.211; Nagelkerke R² = 0.348). Calibration curve analysis (**Figure 4**) further revealed close agreement between the predicted probabilities and observed outcomes, with the optimism-corrected (1,000 bootstrap resamples) curve aligning satisfactorily along the ideal diagonal. The Brier score of the model is 0.1063. At the optimal Youden-derived cut-off of 0.144 (sensitivity 82.9%, specificity 70.0%), the model effectively distinguished patients at elevated risk of postoperative complications. Decision curve analysis (**Figure 5**) showed the combined model yielded higher net benefit than both “treat all” and “treat none” strategies across clinically relevant threshold probabilities, supporting its clinical utility.

**Figure 3:**
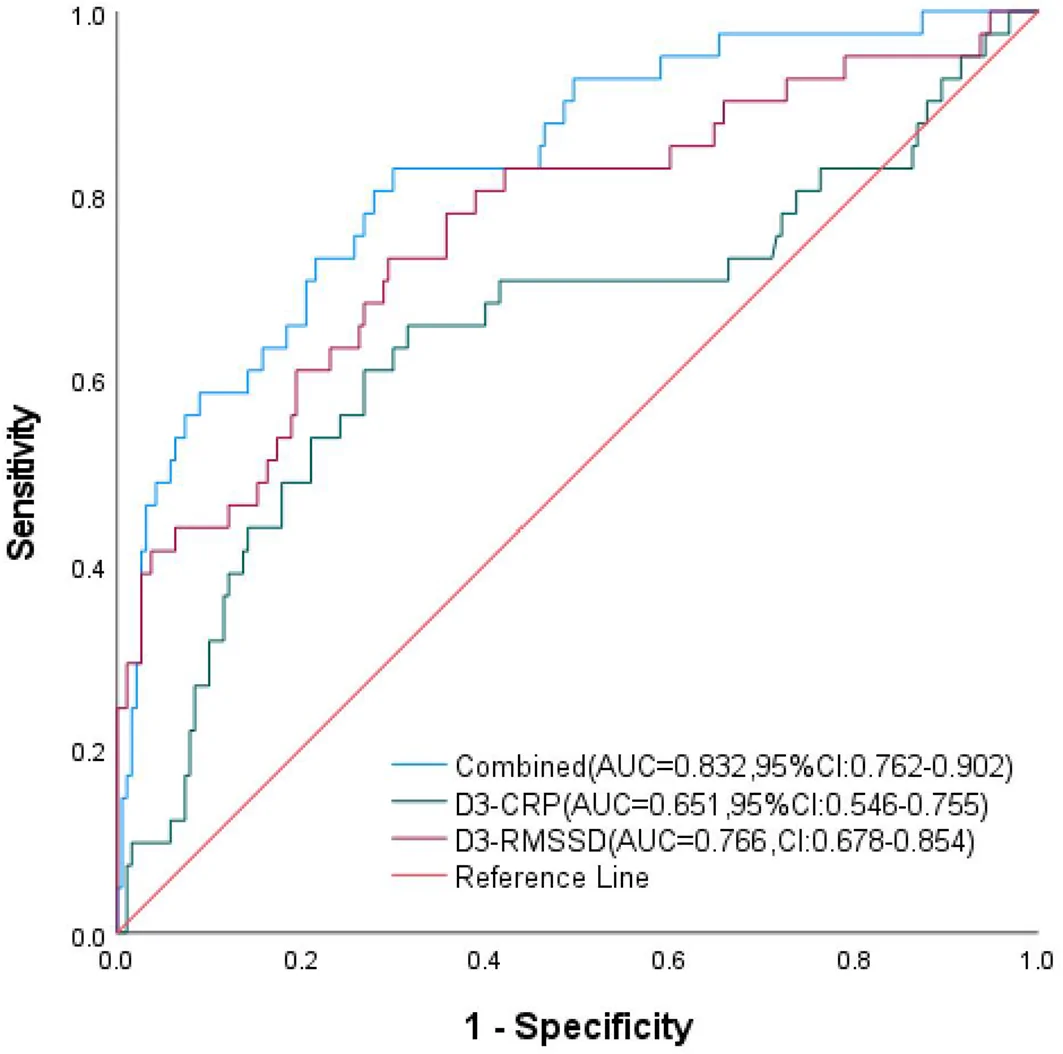
Plot of ROC curve.

**Figure 4:**
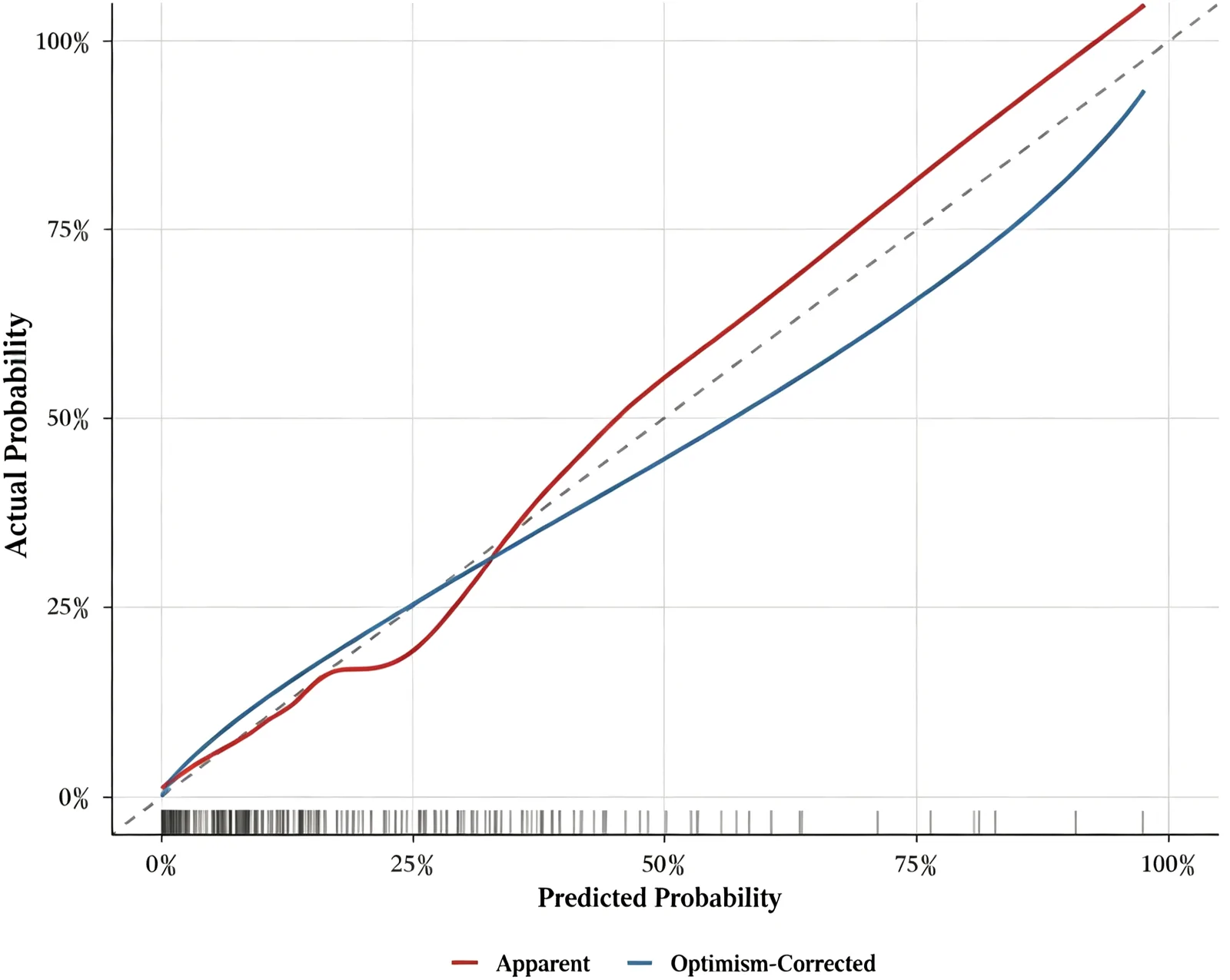
Calibration curve.

**Figure 5:**
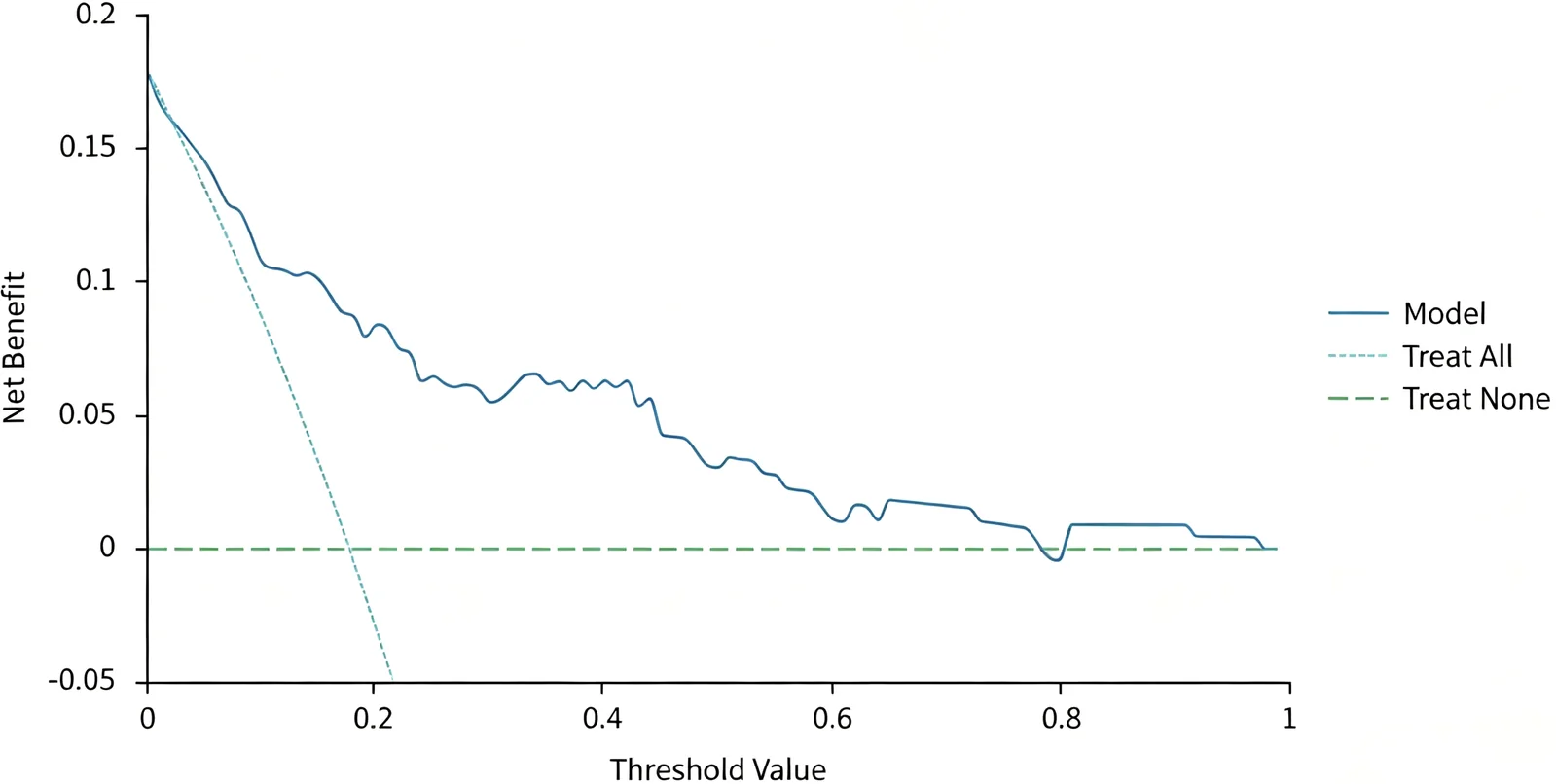
Decision curve analysis.

## Discussion

This study identified RMSSD on postoperative day 3 as an independent protective factor against short-term complications following gastrointestinal cancer surgery (AUC = 0.766, 95% CI: 0.678–0.854, P < 0.001), a finding consistent with the known physiology of autonomic regulation. RMSSD is a validated time-domain marker of cardiac vagal tone that decreases with diminished parasympathetic activity. Gastrointestinal oncologic surgery represents a severe traumatic insult involving bowel resection, digestive tract reconstruction, and potential autonomic nerve injury, all of which can disrupt sympathovagal balance. Vagal withdrawal not only delays gastrointestinal motility recovery and increases the risk of postoperative ileus, but also attenuates the cholinergic anti-inflammatory pathway (CAP), thereby promoting an exaggerated inflammatory response and elevating the risk of infectious complications and anastomotic leakage (19).

In our multivariable analysis, CRP on postoperative day 3 was identified as an independent risk factor (OR = 1.012, 95% CI: 1.003–1.021, P = 0.008), suggesting that its prognostic information may partly reflect the integrated net effect of multiple inflammatory cascades—a parameter routinely assessed in clinical practice. The combined model incorporating both RMSSD and CRP achieved superior discriminative performance (AUC = 0.832) compared with either biomarker alone, indicating that neurophysiological and inflammatory indices provide complementary prognostic value in assessing postoperative physiological status. The simultaneous occurrence of elevated CRP and reduced RMSSD in the complication group suggests a potential interplay involving the neuro-immune-inflammatory axis (20).

The temporal pattern observed in this study further supports this view: HRV parameters in both groups decreased postoperatively relative to preoperative values, reflecting the acute autonomic disturbance induced by surgical trauma. However, whereas the non-complication group exhibited progressive recovery of RMSSD and LF/HF toward baseline by postoperative day 3, the complication group maintained a sustained pattern of sympathetic predominance (elevated LF/HF) and vagal withdrawal (reduced RMSSD). This divergent trajectory suggests that failure of autonomic recovery—rather than the mere presence of an initial stress response—may be a key feature distinguishing uncomplicated from complicated postoperative courses. Previous studies have shown that HRV parameters possess common predictive or monitoring value across various postoperative complications, and that the dynamic recovery trajectory of HRV helps differentiate physiological stress from pathological dysregulation, aligning with the proposal by Shaffer et al. (8). Our findings are consistent with this perspective, as the temporal divergence between groups became apparent only by postoperative day 3, underscoring the importance of continuous monitoring for detecting delayed autonomic recovery.

The pathophysiological patterns underlying different postoperative complications are heterogeneous, and the corresponding autonomic disturbances may likewise exhibit distinct characteristics. Common severe postoperative complications include postoperative hemorrhage, anastomotic leak, and postoperative ileus (POI). Postoperative hemorrhage is associated with excessive sympathetic activation and decompensation. Although initial sympathetic activation may compensate for blood loss, persistent activation may lead to visceral ischemia and delayed hemostasis (21, 22). If hemorrhage progresses, HRV may shift toward overall suppression, a pattern that has been associated with circulatory failure (23, 24). For anastomotic leak and subsequent sepsis, vagal withdrawal and sympathetic hyperactivity may contribute to weakened immune defense and excessive inflammation (24–26), impairing tissue repair and increasing infection risk (27–29). Observational studies have reported that the degree of HRV decrease is associated with the severity and mortality among patients with sepsis (30, 31). For POI, its occurrence has been linked to decreased vagal tone, as may be reflected by HRV (31). Improvements in HRV parameters following intervention have been associated with vagal reactivation and gastrointestinal functional recovery (32–34).

Current perioperative risk assessment still relies largely on static indicators and traditional models, with insufficient attention to autonomic nervous function (19). Our findings suggest that incorporating dynamic HRV monitoring into ERAS protocols may help identify patients with autonomic dysfunction, thereby informing individualized interventions such as enhanced prehabilitation (35) and refined fluid management (36, 37), with the potential to shorten hospital stays and reduce complication rates (38, 39).

However, HRV parameters are highly sensitive to physiological and environmental factors, including circadian rhythms, physical activity, pain, and pharmacological agents; rigorous measurement standardization is therefore essential. Moreover, although the cholinergic anti-inflammatory pathway provides a plausible framework for interpreting our findings, this study measured only peripheral inflammatory biomarkers and did not directly assess pathway-specific mediators such as α7 nicotinic acetylcholine receptor expression or vagal efferent activity. Consequently, neuroimmune interactions should be regarded as a biologically reasonable hypothesis derived from our data rather than a confirmed causal mechanism. Direct verification will require targeted experimental approaches, including cytokine-specific assays or direct electrophysiological recordings.

Several limitations should be acknowledged. First, because this was a retrospective study, data completeness relied on medical records, and potential confounding factors such as preoperative nutrition and anesthetic medication were not fully controlled, which may affect the accuracy of association analyses (9). Second, the sample size may still be insufficient for statistical power regarding rare complications like anastomotic leak and major postoperative hemorrhage. Third, the single-center design results in relatively homogeneous patient sources, treatment protocols, and surgical teams, limiting the generalizability of the conclusions, which requires validation with multicenter external data.

## Conclusion

In this retrospective cohort study, dynamic perioperative heart rate variability monitoring, particularly the RMSSD on postoperative day 3, was significantly associated with the risk of short-term complications following gastrointestinal cancer surgery. The combination of RMSSD with CRP provided complementary prognostic information and improved risk discrimination compared with either biomarker alone. The observed co-occurrence of reduced vagal tone and inflammatory activation is compatible with a potential involvement of neuroimmune interactions, although this mechanistic interpretation remains speculative and requires direct experimental validation. These findings support the potential utility of HRV as a non-invasive, continuously obtainable physiological biomarker for perioperative risk stratification. However, given the retrospective, single-center design and the exploratory nature of these analyses, the results should be interpreted with caution. Prospective, multicenter studies with larger sample sizes are warranted to validate the generalizability of these observations and to establish the clinical efficacy of HRV-guided interventions within enhanced recovery protocols.

## Data Availability

The raw data supporting the conclusions of this article will be made available by the authors, without undue reservation.

## References

[1] Bray F Laversanne M Sung H Ferlay J Siegel RL Soerjomataram I . Global cancer statistics 2022: GLOBOCAN estimates of incidence and mortality worldwide for 36 cancers in 185 countries. CA: A Cancer J For Clin. (2024) 74:229–63. doi: 10.3322/caac.21834 38572751

[2] Iyer S Saunders WB Stemkowski S . Economic burden of postoperative ileus associated with colectomy in the United States. J Managed Care Pharmacy: JMCP. (2009) 15:485–94. doi: 10.18553/jmcp.2009.15.6.485 19610681 PMC10438346

[3] Zhou Y Yin ZH Sun MS Wang YY Yang C Li SH . Global research trends in postoperative ileus from 2011 to 2023: A scientometric study. World J Gastrointestinal Surg. (2024) 16:3020–31. doi: 10.4240/wjgs.v16.i9.3020 39351552 PMC11438810

[4] van Hootegem SJM van der Linde M Schneider MA Kim J Berlth F Sugita Y . Impact of postoperative complications on clinical outcomes after gastrectomy for cancer: multicentre study. Br J Surg. (2025) 112:znaf043. doi: 10.1093/bjs/znaf043 40156166 PMC11953074

[5] Sivakumar J Alnimri F Johnson MA Ward S Chong L Hii MW . Health economic analysis of curative-intent gastrectomy for gastric carcinoma and the costs related to post-operative complications. Anz J Surg. (2021) 91:E1–6. doi: 10.1111/ans.16014 32483869

[6] Traeger L Koullouros M Bedrikovetski S Kroon HM Thomas ML Moore JW . Cost of postoperative ileus following colorectal surgery: A cost analysis in the Australian public hospital setting. Colorectal Dis. (2022) 24:1416–26. doi: 10.1111/codi.16235 35737846 PMC9796387

[7] Bedford JP Redfern OC O'Brien B Watkinson PJ . Perioperative risk scores: prediction, pitfalls, and progress. Curr Opin Anaesthesiology. (2025) 38:30–6. doi: 10.1097/ACO.0000000000001445 PMC11676592 39526674

[8] Shaffer F Ginsberg JP . An overview of heart rate variability metrics and norms. Front Public Health. (2017) 5:258. doi: 10.3389/fpubh.2017.00258 29034226 PMC5624990

[9] Reimer P Máca J Szturz P Jor O Kula R Ševčík P . Role of heart-rate variability in preoperative assessment of physiological reserves in patients undergoing major abdominal surgery. Ther Clin Risk Manage. (2017) 13:1223–31. doi: 10.2147/tcrm.s143809 29033572 PMC5614745

[10] Frandsen MN Mehlsen J Foss NB Kehlet H . Preoperative heart rate variability as a predictor of perioperative outcomes: a systematic review without meta-analysis. J Clin Monit Comput. (2022) 36:947–60. doi: 10.1007/s10877-022-00819-z 35092527 PMC9293802

[11] Cheng W Jiang ZW . Digitalization of perioperative traumatic stress in enhanced recovery after surgery program: current application and future prospect. Zhonghua Wei Chang Wai Ke Za Zhi = Chin J Gastrointestinal Surg. (2022) 25:575–81. doi: 10.3760/cma.j.cn441530-20220324-00115 35844119

[12] Pache B Hübner M Jurt J Demartines N Grass F . Minimally invasive surgery and enhanced recovery after surgery: The ideal combination? J Surg Oncol. (2017) 116:613–6. doi: 10.1002/jso.24787 29081065

[13] Pei L Wang G Yang S Zhou S Xu T Zhou J . Electroacupuncture reduces duration of postoperative ileus after laparoscopic gastrectomy for gastric cancer: A multicenter randomized trial. Gastroenterology. (2025) 169:73–84. doi: 10.1053/j.gastro.2025.02.006 39978558

[14] Kong Q Chen LM Liu CY Li W Yin PH . The effect of acupuncture on gastrointestinal recovery after abdominal surgery: a narrative review from clinical trials. Int J Surg (London England). (2024) 110:5713–21. doi: 10.1097/js9.0000000000001641 38759698 PMC11392097

[15] Fang Y Zhang Y Wang G Guan J Wang H Shao M . Autonomic function effects on postoperative sleep disorder: a prospective cohort study. Int J Surg. (2025) 111:7010–20. doi: 10.1097/js9.0000000000002630 40956174 PMC12527782

[16] Clavien PA Barkun J de Oliveira ML Vauthey JN Dindo D Schulick RD . The Clavien-Dindo classification of surgical complications: five-year experience. Ann Surg. (2009) 250:187–96. doi: 10.1097/SLA.0b013e3181b13ca2 19638912

[17] Breitenstein S DeOliveira ML Raptis DA Slankamenac K Kambakamba P Nerl J . Novel and simple preoperative score predicting complications after liver resection in noncirrhotic patients. Ann Surg. (2010) 252:726–34. doi: 10.1097/sla.0b013e3181fb8c1a 21037427

[18] Singer M Deutschman CS Seymour CW Shankar-Hari M Annane D Bauer M . The third international consensus definitions for sepsis and septic shock (Sepsis-3). JAMA. (2016) 315:801–10. doi: 10.1001/jama.2016.0287 26903338 PMC4968574

[19] Pan WT Ji MH Ma D Yang JJ . Effect of perioperative autonomic nervous system imbalance on surgical outcomes: a systematic review. Br J Anaesthesia. (2025) 135:608–22. doi: 10.1016/j.bja.2025.06.004 40615330 PMC12489373

[20] Haensel A Mills PJ Nelesen RA Ziegler MG Dimsdale JE . The relationship between heart rate variability and inflammatory markers in cardiovascular diseases. Psychoneuroendocrinology. (2008) 33:1305. doi: 10.1016/j.psyneuen.2008.08.007 18819754 PMC4266571

[21] Hao J Li J Zhang H Chen J Fang M Wu M . Gastrointestinal bleeding risk factors in type A aortic dissection post-aortic arch replacement. J Thorac Dis. (2024) 16:2314–25. doi: 10.21037/jtd-23-1752 38738230 PMC11087617

[22] Ushiyama T Mizushige K Wakabayashi H Nakatsu T Ishimura K Tsuboi Y . Analysis of heart rate variability as an index of noncardiac surgical stress. Heart Vessels. (2008) 23:53–9. doi: 10.1007/s00380-007-0997-6 18273547

[23] Ushiyama T Nakatsu T Yamane S Tokutake H Wakabayashi H Ishimura K . Heart rate variability for evaluating surgical stress and development of postoperative complications. Clin Exp Hypertension (New York NY: 1993). (2008) 30:45–55. doi: 10.1080/10641960701813908 18214733

[24] Liu Z Jian C Yuan W Jia G Cheng D Liu Y . Epinephrine promotes tumor progression and M2 polarization of tumor-associated macrophages by regulating the TRIM2- NF-κB pathway in colorectal cancer cells. Genes Dis. (2024) 11:101092. doi: 10.1016/j.gendis.2023.101092 38515938 PMC10955210

[25] Straub RH Pongratz G Weidler C Linde HJ Kirschning CJ Glück T . Ablation of the sympathetic nervous system decreases gram-negative and increases gram-positive bacterial dissemination: key roles for tumor necrosis factor/phagocytes and interleukin-4/lymphocytes. J Infect Dis. (2005) 192:560–72. doi: 10.1086/432134 16028124

[26] Kiryachkov YY Bosenko SA Muslimov BG Petrova MV . Dysfunction of the autonomic nervous system and its role in the pathogenesis of septic critical illness (Review). Sovremennye Tekhnologii V Meditsine. (2021) 12:106–16. doi: 10.17691/stm2020.12.4.12 PMC8596275 34795998

[27] Kushioka J Chow SKH Toya M Tsubosaka M Shen H Gao Q . Bone regeneration in inflammation with aging and cell-based immunomodulatory therapy. Inflammation Regeneration. (2023) 43:29. doi: 10.1186/s41232-023-00279-1 37231450 PMC10210469

[28] Wang Chau C Sugimura R . Locked in a pro-inflammatory state. eLife. (11) 11:e80699. doi: 10.7554/eLife.80699 PMC9262384 35796529

[29] Tuleta I Frangogiannis NG . Diabetic fibrosis. Biochim Biophys Acta Mol Basis Dis. (2021) 1867:166044. doi: 10.1016/j.bbadis.2020.166044 33378699 PMC7867637

[30] de Castilho FM Ribeiro ALP Nobre V Barros LC Ribeiro AL . Heart rate variability as predictor of mortality in sepsis: A systematic review. PloS One. (2018) 13:e0203487. doi: 10.1371/journal.pone.0203487 30204803 PMC6133362

[31] Brandlhuber M Benhaqi P Brandlhuber B Koliogiannis V Kasparek MS Mueller MH . The role of vagal innervation on the early development of postoperative ileus in mice. Neurogastroenterol Motil. (2022) 34:e14308. doi: 10.1111/nmo.14308 34962331

[32] Stakenborg N Wolthuis AM Gomez-Pinilla PJ Farro G Di Giovangiulio M Bosmans G . Abdominal vagus nerve stimulation as a new therapeutic approach to prevent postoperative ileus. Neurogastroenterol Motil. (2017) 29:e13075. doi: 10.1111/nmo.13075 28429863

[33] Ru O Jin X Qu L Long D Liu M Cheng L . Low-intensity transcutaneous auricular vagus nerve stimulation reduces postoperative ileus after laparoscopic radical resection of colorectal cancer: a randomized controlled trial. Minerva Anestesiologica. (2023) 89:149–56. doi: 10.23736/s0375-9393.22.16735-0 36326770

[34] Cai Y Li H Nan H Xu P Li J Pan H . ASO visual abstract: Randomized trial on electroacupuncture for recovery of postoperative gastrointestinal function based on long-term monitoring device. Ann Surg Oncol. (2025) 32:6916–. doi: 10.1245/s10434-025-17415-5 PMC12129847 40183888

[35] Oliveira NL Ribeiro F Alves AJ Teixeira M Miranda F Oliveira J . Heart rate variability in myocardial infarction patients: effects of exercise training. Rev Portuguesa Cardiologia: Orgao Oficial Da Sociedade Portuguesa Cardiologia = Portuguese J Cardiology: Off J Portuguese Soc Cardiol. (2013) 32:687–700. doi: 10.1016/j.repc.2013.02.010 23993292

[36] Bodenes L N'Guyen QT Le Mao R Ferrière N Pateau V Lellouche F . Early heart rate variability evaluation enables to predict ICU patients’ outcome. Sci Rep. (2022) 12:2498. doi: 10.1038/s41598-022-06301-9 35169170 PMC8847560

[37] Malbrain MLNG Marik PE Witters I Cordemans C Kirkpatrick AW Roberts DJ . Fluid overload, de-resuscitation, and outcomes in critically ill or injured patients: a systematic review with suggestions for clinical practice. Anaesthesiology Intensive Ther. (2014) 46:361–80. doi: 10.5603/ait.2014.0060 25432556

[38] Aagaard N Olsen MH Rasmussen OW Grønbaek KK Mølgaard J Haahr-Raunkjaer C . Prognostic value of heart rate variability for risk of serious adverse events in continuously monitored hospital patients. J Clin Monit Comput. (2024) 38:1315–29. doi: 10.1007/s10877-024-01193-8 39162840 PMC11604769

[39] Chiew CJ Liu N Tagami T Wong TH Koh ZX Ong MEH . Heart rate variability based machine learning models for risk prediction of suspected sepsis patients in the emergency department. Medicine. (2019) 98:e14197. doi: 10.1097/md.0000000000014197 30732136 PMC6380871

